# The physicochemical properties of chitosan prepared by microwave heating

**DOI:** 10.1002/fsn3.1486

**Published:** 2020-03-02

**Authors:** Jiaqi Cheng, Huaping Zhu, Jianlian Huang, Jianxin Zhao, Bowen Yan, Shenyan Ma, Hao Zhang, Daming Fan

**Affiliations:** ^1^ State Key Laboratory of Food Science and Technology Jiangnan University Wuxi China; ^2^ School of Food Science and Technology Jiangnan University Wuxi China; ^3^ China Rural Technology Development Center Beijing China; ^4^ Key Laboratory of Refrigeration and Conditioning Aquatic Products Processing Ministry of Agriculture and Rural Affairs Xiamen China; ^5^ Fujian Anjoyfood Share Co. Ltd. Xiamen China

**Keywords:** chitosan, deacetylation degree, microwave, physicochemical properties, water bath

## Abstract

The aim of this study was to compare the physicochemical properties of chitosan prepared by microwave and water bath heating with an equivalent quantity of heat intake. The structure and physicochemical properties of the chitosan obtained by these two methods were characterized by Fourier transform infrared spectroscopy (FTIR), X‐ray diffractometry (XRD), gel permeation chromatography (GPC), and scanning electron microscopy (*SEM*). The FTIR and XRD patterns show that there was no significant difference in the structure of chitosan produced by the two heat sources. The results showed that chitosan with 73.86% deacetylation was successfully prepared by microwave heating within 60 min, while a longer time of 180 min was required for the preparation of chitosan with the same deacetylation degree (74.47%) using the conventional heating method under the same heating rate. Even under the same temperature conditions, microwave technology can greatly reduce the reaction time by approximately 1/3, while the chitosan produced by microwaves can obtain relatively low molecular weight and viscosity. These results showed that microwaves may efficiently promote complete chemical reactions by the friction heating mechanism generated by molecular vibration beyond a rapid heating source, turning into a more efficient, energy‐saving, and environmentally friendly method for the further use of rigid shrimp shells and highly crystalline crustacean materials.

## Graphical abstract

The physicochemical properties of chitosan prepared by microwave and water bath heating were compared with an equivalent quantity of heat intake. There was no significant difference in the structure of chitosan produced by the two heat sources. Microwave technology can greatly reduce the reaction time by approximately 1/3, while the chitosan produced by microwaves can obtain relatively low molecular weight and viscosity.

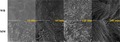

## 1 INTRODUCTION

Chitin, an important natural polysaccharide, is the most abundant natural polymer in nature after cellulose (Younes & Rinaudo, 2015). It is usually found in the exoskeletons of arthropods or in the cell walls of fungi and yeast, and its main commercial source is shrimp and crab shells (Croisier & Jérôme, 2013). Chitin is covalently bound to protein in the form of proteoglycan in shrimp and crab shells, accompanied by layers of calcium carbonate, and the quality of chitin extracted from shrimp and crabs at different growth stages by the same method is quite different (Omari, Besaw, & Kerton, 2012). The heterogeneity of chitin caused by differences in raw materials is a hindrance for the application of chitin and its derivatives.

Chitosan is a derivative of chitin formed by deacetylation under alkaline conditions; when the degree of deacetylation (DD) of chitin is above 50%, it is called chitosan and can be dissolved in acidic aqueous solutions (Rinaudo, 2008). However, 100% deacetylation cannot be achieved; therefore, chitosan is a copolymer of N‐acetylglucosamine and glucosamine which exhibits outstanding biocompatibility, biodegradability, and adsorbability.

At present, different methods have been proposed to prepare chitosan from chitin, mainly by using a chemical process, highly concentrated sodium hydroxide solution at high temperature (Mohammed, Williams, & Tverezovskaya, 2013), or the enzymatic deacetylation method (Jaworska & Roberts, 2016; Liu et al., 2019). Ma, Xin, and Tan (2015) have attempted to repeatedly immerse chitin in alkali solution due to difficulty of obtaining a high DD in one round of high‐temperature lye treatment. However, this method is time‐consuming and prone to cause environmental pollution. Thus, physical means have been introduced to promote deacetylation, such as high pressure (He et al., 2016), ultrasound (Zhu et al., 2019), and microwave (MW) (Sebastian, Rouissi, Brar, Hegde, & Verma, 2019). Knidri, Dahmani, Addaou, Laajeb, and Lahsini (2019) found that chitin deacetylation by MW irradiation was more efficient than the customary heating method, and a high DD can be obtained by utilizing MW for a few minutes. The principle of MW heating is by producing an electromagnetic field that causes vibration within the molecules of a material (Abeykoon, Kelly, Brown, & Coates, 2016), and it offers increased reaction rates and shorter reaction times; therefore, a higher DD can be obtained in a shorter time by using the rapid heating rate. Previous researchers improved the extraction rate of chitosan by setting a certain MW power to obtain rapid heating, reducing the extraction time from hours to minutes (Alishahi et al., 2011; Mahardika, Jumnahdi, & Widyaningrum, 2019), but whether electromagnetic heating will affect the quality of chitosan, except for the difference in the heating rate, has not been studied.

This work proposed to compare the physicochemical properties of chitosan prepared from chitin with MW technology and traditional methods at the same heating rate to provide the equivalent heat quantity by fitting the traditional water bath (WB) heating curve and matching the corresponding MW program while comparing the efficiency of the two methods.

## 2 MATERIALS AND METHODS

### 2.1 Materials

Commercial chitin and chitosan (80% degree of deacetylation) were purchased from TCI. Sodium hydroxide, hydrochloric acid, glacial acetic acid, and ammonium acetate were purchased from Sinopharm Group Co. Ltd. All of the reagents used were of analytical grade, and ultrapure water was used for preparation of all solutions (electrical conductivity: 1.51 × 10^–4^ S/m).

### 2.2 Methods

In this study, the superiority of MW heating in the extraction of chitosan was determined by comparing the quality of the final products produced by the two methods, which were controlled at the same heating rates. Conventional heating was compared with MW heating in all processes.

#### 2.2.1 Preparation of chitosan

The traditional deacetylation conversion of chitin to chitosan was performed using the method suggested by Sarhan et al. with slight modification (Sarhan, Ayad, Badawy, & Monier, 2009). The parameters employed were as follows: a suspension of 1 g chitin in 30 ml 50% sodium hydroxide at 90°C WB under constant stirring for 4 hr. During the 4‐hr deacetylation process, in order to study the physicochemical properties of samples at different reaction stages sampling started after 10 min. After deacetylation, the solid was filtered on a 240‐mesh sifter and washed with water until the filtrate was neutral. Then, it was freeze‐dried for 48 hr.

The preparation of chitosan by MW irradiation was carried out using a MW reaction device (flexiWAVE, MILESTONE). The reaction parameters were consistent with the traditional method mentioned above, especially the heating rate. The temperature of WB heating measured by optical fiber was recorded; then, the data were sent to the MW equipment which adjusts the power to meet the corresponding temperature demand.

Chitosan produced under MW or WB heating is noted as chitosan MW and chitosan WB, respectively.

#### 2.2.2 Characterization of chitin and chitosan

##### Fourier transform infrared spectroscopy (FTIR)

To obtain uniform samples, chitosan samples were dissolved in 1% acetic acid solution, dried into a film, neutralized with 0.1 m sodium hydroxide solution, washed, and dried again. The FTIR spectra of chitin, chitosan, and commercial samples were recorded at 4 cm^−1^ spectral resolution on a FTIR spectrometer (Nicolet Nexus 470, Thermo Electron) equipped with a horizontal attenuated total reflectance (HATR) accessory (Thermo Electron). An average of 32 scans was performed for each spectrum. The DD% of chitosan was determined by the absorption bands at 1,320 cm^−1^ (amide III band) and 1,420 cm^−1^ proposed by Brugnerotto et al. (2001), knowing that:

DD%=100-DA%

The DA% is the degree of acetylation calculated according to the following equation:

(1) $$A_{1320}/A_{1420}=0.3822+0.0313\;\text{DA}$$

where *A*_1320_ and *A*_1420_ are the absorbance at 1,320 cm^−1^ and 1,420 cm^−1^ with baselines drawn between the 1370–1280 cm^−1^ and 1490–1350 cm^−1^, respectively.

##### XRD

X‐ray diffractograms of the samples were obtained using a Bruker X‐ray diffractometer (D2 PHASER, Bruker AXS Inc.) with 40 kV and 30 mA Cu kα radiation at a scanning rate of 2°min^−1^ between 2θ angles from 5° to 40°. The crystallinity index I_CR_ was calculated by the following equation (Ioelovich, 2014):

(2) $$I_{\text{CR}}\%=\left[\left(I_{110}-I_{\text{am}}\right)/I_{110}\right]\times 100$$

where *I*_am_ is the intensity of amorphous diffraction at 16°, and *I*_110_ is the maximum intensity at 20°.

##### Molecular weight determination

The weight‐average molecular weight (M_W_) of chitosan was measured by gel permeation chromatography (GPC) (Li & Xia, 2010). The GPC equipment (Wyatt) consisted of an Ultrahydrogel 2000 (7.8 mm × 300 mm) combined with an Ultrahydrogel 250 (7.8 mm × 300 mm), an RI 150 refractive index detector, and a Waters 600 Pump. The eluent was 0.2 M CH_3_COOH/0.15 M CH_3_COONH_4_. The temperature of the column and the flow rate were maintained at 303 K and 0.4 ml/min, respectively. Chitosan samples were prepared with the same acetate buffer at a concentration of 1 mg/ml, and dissolved samples were filtered through a 0.22‐μm filter. All data provided by the GPC system were collected and analyzed using the Waters Workstation software package.

##### Scanning electron microscopy (*SEM*)

For the visual confirmation of the morphology and physical state of the samples, a Hitachi Regulus 8,100 *SEM* instrument was used. The freeze‐dried samples were glued to the sample stage with conductive adhesive. The images of the samples were taken at different magnifications and an acceleration voltage of 5 kV.

#### 2.2.3 Determination of rheological properties

The rheological properties of chitosan solutions were measured by an AR 1,000 rheometer (TA Instrument Ltd.). The sample chitosan (1 g) was added into 1% acetic acid solution (100 ml) and then stirred until completely dissolved. The viscosity of the sample chitosan at different shear rates from 0.1 to 100 s^−1^ was measured with a 40‐mm parallel plate at a spacing of 1 mm (Li et al., 2019). TA Rheometer Data Analysis software (V1.0.74) was used to obtain the experimental data.

#### 2.2.4 Statistical analysis

The experimental data are represented as the mean ± standard deviations. Statistical analysis was performed using IBM SPSS Statistics 19.0 for Windows (SPSS Inc.). Multiple comparisons of mean values were conducted using Duncan's multiple range test. The *p* value >.05 indicated no significant differences between the mean values.

## 3 RESULTS AND DISCUSSION

### 3.1 Fitting of heating rate curves

**Figure 1:**
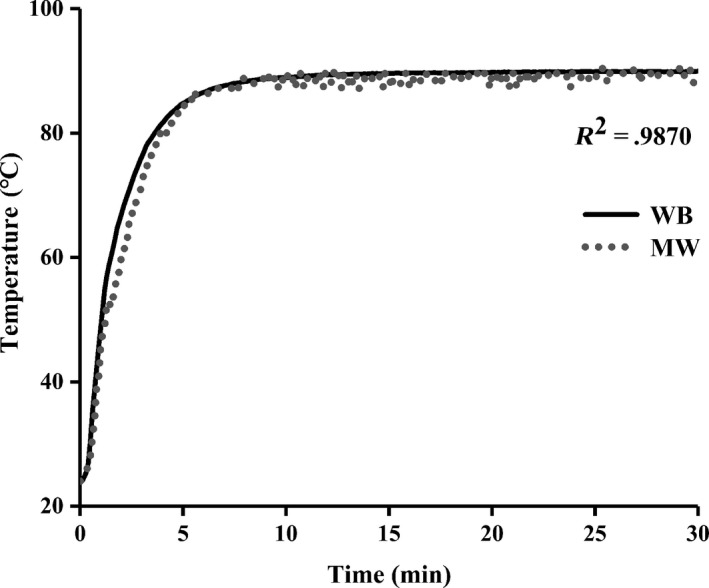
Heating curves for microwave heating and water bath heating

In previous studies, many researchers have used MW instead of WB heating in the conventional method of chitosan extraction and found that by setting high MW power, a better extraction effect can be achieved in a short time, but the temperature difference between the two methods was ignored (El Knidri, El Khalfaouy, Laajeb, Addaou, & Lahsini, 2016). In this study, a multistage MW heating program was used to simulate the heating curve of a WB in the process of deacetylation. The effects of WB and MW on the extraction of chitosan were compared under the same heating rate, and the heating curves of the two heating methods are shown in Figure 1. The correlation coefficient *R* ^2^ was used to evaluate the fitting degree of the two heating curves. The *R* ^2^ value calculated by Excel software for the deacetylation step is 0.9870. In this study, the value can represent the difference of the average temperature of MW and WB heating, so it can be seen that the heating curves obtained by the two heating methods had a high degree of fitting. This ensured the consistency of the heat quantity provided by the two heating methods and the comparability of the differences between chitosan MW and chitosan WB.

### 3.2 FTIR patterns and deacetylation degree analysis

**Figure 2:**
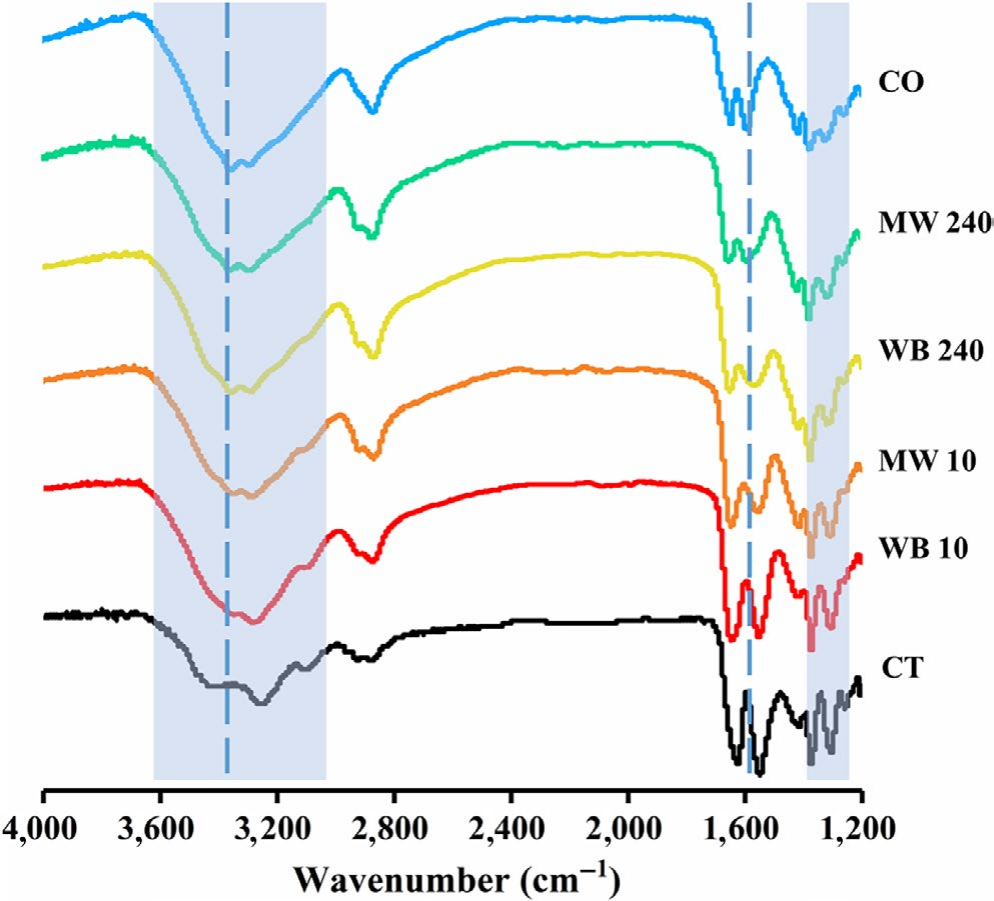
FTIR patterns of commercial chitin CT, commercial chitosan CO, MW10, WB10, MW240, and WB240

Fourier transform infrared spectroscopy is an important tool to differentiate chitin and chitosan. During the reaction process, the chitosan MW and chitosan WB produced in different time periods were compared. Commercial chitin, commercial chitosan, and the chitosan produced by MW or WB in 10 min (initial deacetylation) and 240 min (completed deacetylation), denoted as CT, CO, MW10, WB10, MW240, and WB240, respectively, exhibit different FTIR spectra from 1,200 to 4,000 cm^−1^. In Figure 2, the bands at approximately 3,100 and 1,640 cm^−1^ were assigned to amide I (Kaya, Erdogan, Mol, & Baran, 2015), while those at 1,550 and 1,310 cm^−1^ were assigned to amides II and III, respectively, in CT (Kaya & Baran, 2015; Kaya, Baran, Erdoğan, et al., 2014). The absorption of these bands weakens or disappears in chitosan WB and chitosan MW in the progress of deacetylation, confirming the removal of the acetyl group. Corresponding to the NH stretching region (Chatterjee, Adhya, Guha, & Chatterjee, 2005) of chitosan, the absorption bands in the range of 3270–3290 cm^−1^ were observed in all samples, but their intensity in CT was relatively weaker. The peak at 1599 cm^−1^ of the amide II (‐NH_2_ tensions), which is the characteristic peaks for chitosan (Kaya, Baran, Mentes, et al., 2014), in the CO, MW240, and WB240 spectra was absent in the spectra of CT, MW10, and WB10. The FTIR results showed that the chitosan produced by MW and WB after deacetylation for 240 min had a structure similar to that of commercially available chitosan.

**Figure 3:**
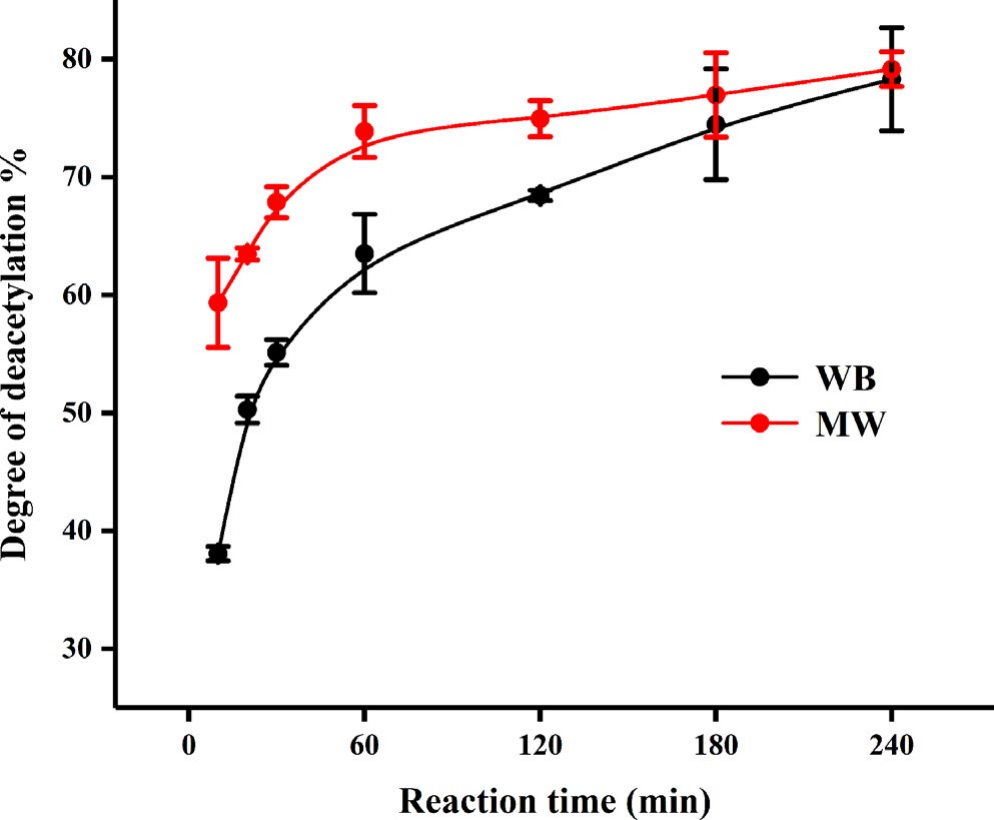
Deacetylation degree of chitosan produced by microwave heating and water bath heating

The DD% was calculated using the ratio of the bands at 1,420 and 1,320 cm^−1^ according to the FTIR patterns (Figure 2), as described in Equation (1). The chitosan MW with 73.86% DD was successfully produced in 60 min, while 120 more minutes were required for the same DD% (74.47%) of the chitosan WB. Additionally, it can be seen from Figure 3 that the MW heating method basically completed the deacetylation reaction at 60 min, and the DD% value increased slightly with the extension of time, while the traditional method needed 3–4 hr to complete the reaction.

**Figure 4:**
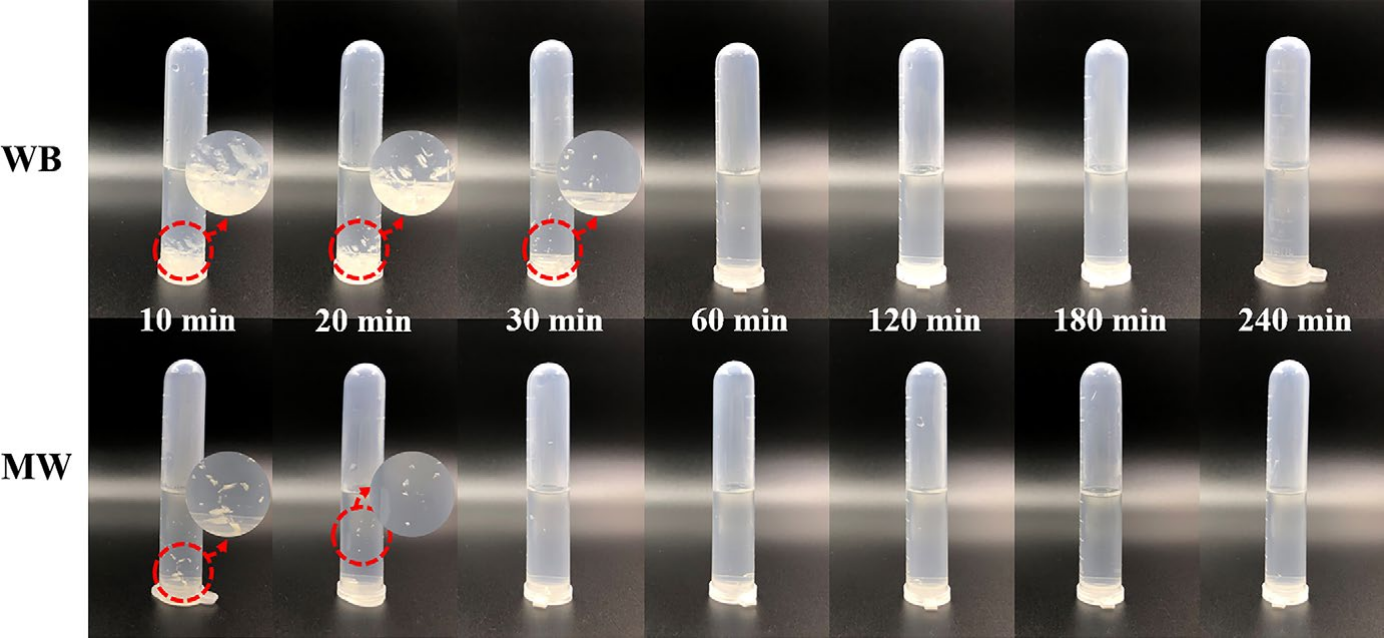
Digital photographs of chitosan WB and chitosan MW in the 1% acetic acid solution

The solubility of chitosan in 1% acetic acid solution mainly depends on the DD% value and on the acetyl group distribution along the chains (Roy et al., 2017; Younes & Rinaudo, 2015). Chitin with a DD% above 55% can be solubilized gradually. Chitosan WB can be basically dissolved after 60 min, as shown in Figure 4, while chitosan MW can be basically dissolved after deacetylation for 30 min, which was consistent with the results of DD%.

### 3.3 XRD pattern analysis

**Figure 5:**
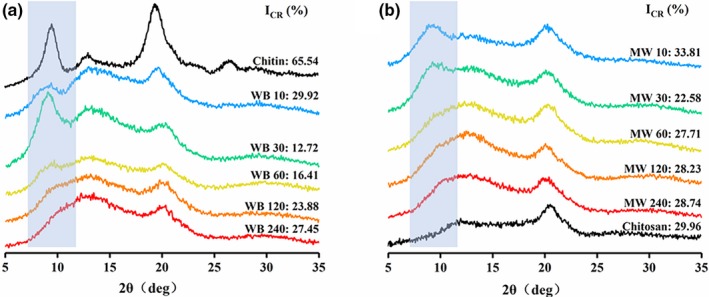
XRD patterns of commercial chitin, chitosan WB (a), commercial chitosan, and chitosan MW (b)

The XRD patterns and I_CR_% of commercially available chitin and chitosan, the chitosan produced by MW or WB for 10 min, 30 min, 60 min, 120 min, and 240 min (denoted as MW10, WB10, MW30, WB30, and so on, respectively), are presented in Figure 5. The spectra of chitosan WB and chitosan MW have the same positions of peaks (2θ = 11.92–12.29° and 2θ = 20.01–20.56°) as the commercial chitosan, concurring with the results given by Yen, Yang, and Mau (2009 and Zhang et al. (2017), although one method took 240 min, while the other required 60 min.

The crystallinity of chitosan has a high correlation with its DD. The molecular chain of nondeacetylated chitin is relatively uniform and has good regularity, so the crystallinity is high. Deacetylation causes heterogeneity of the molecular chain so that crystallinity decreases; however, as DD increases, the molecular chain tends to become homogenized, and its crystallinity also increases accordingly. With the progress of the deacetylation reaction, the change in the crystallinity of chitosan WB first decreased to 12.72% at 30 min and then rose gradually, corresponding to the molecular chain order changing to disorder and then to order, which was the same as chitosan MW, which dropped to 22.58% at 30 min and then rose to 27.71% at 60 min. In the reaction process, chitosan MW showed relatively high crystallinity, which corresponded to a more ordered molecular chain arrangement, indicating that MW could promote the uniformity and thoroughness of the deacetylation reaction.

### 3.4 Molecular weight and viscosity

M_W_ determines the functional properties of chitosan as it considerably affects the physicochemical properties (Czechowska‐Biskup, Wach, Rosiak, & Ulański, 2018), so M_W_ is one of the most important parameters of chitosan. Generally, deacetylation is accompanied by a decrease in M_W_ because in high concentrations of alkali solution, in addition to the shedding of N‐acetyl groups on glucosamine, the β‐1,4 glycosidic bonds between the disaccharide units in the chitin chain are also broken (Berger et al., 2005).

**Figure 6:**
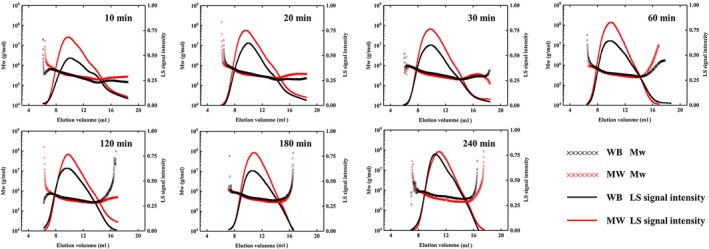
LS signal intensity and the molecular weight distribution corresponding to the elution volume for chitosan samples

The M_W_ distribution corresponding to the elution volume of samples and LS signal intensity are shown in Figure 6. The LS signal intensity characterized the sample content distribution under different elution volumes. From a relative height, the peak of chitosan MW always maintained a higher signal intensity than that of chitosan WB, which proved that chitosan MW had better solubility than chitosan WB in the same solid–liquid solution. At the same time, the span of the molecular weight distribution can be learned from the width of the peak shape of the LS signal intensity and the trend of the M_W_ dot pattern. The M_W_ distribution span of both chitosan MW and chitosan WB gradually narrowed from 10 to 240 min because the shedding of the reaction started with the surface fibers of the chitin, resulting in M_W_ distribution differences in the initial reaction stage.

**Figure 7:**
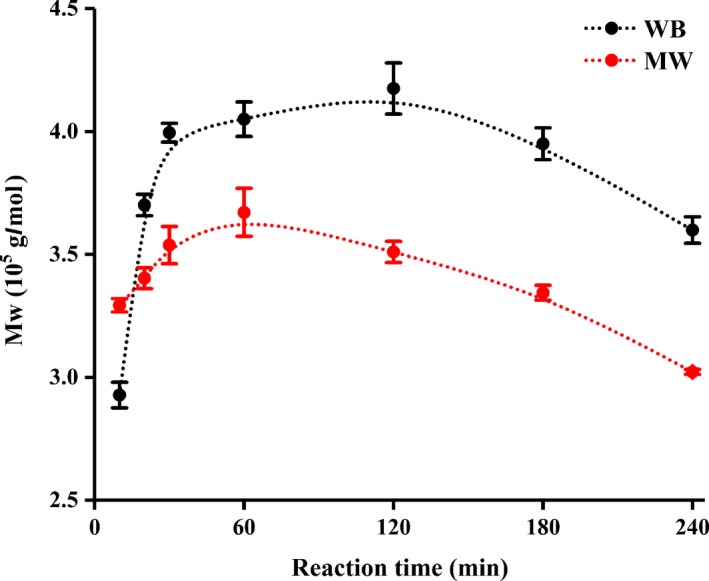
Molecular weight of chitosan produced by microwave heating and water bath heating

The average M_W_ of chitosan produced by the two methods is shown in Figure 7. The M_W_ of both chitosan MW and chitosan WB presented a trend of increasing first and then decreasing, which was due to the average deacetylation degree of the chitin with high M_W_ being relatively low in a short time, so it was difficult to dissolve. Some of the small molecules broken off of the chitin chain showed better solubility after rapid deacetylation, so they could enter the gel column and present their corresponding LS signals.

Generally, the difference in the Mw is caused by the difference in the deacetylation degree, the different sources of chitosan, and several factors in the preparation of chitosan. Chitosan MW and chitosan WB were produced under the same conditions and had a close degree of deacetylation after 240 min of reaction (MW240: 79.15% DD; WB240: 78.29% DD), but the Mw of MW240 (3.022 × 10^5^ g/mol) was lower than that of WB240 (3.599 × 10^5^ g/mol). This may be because MW heating through the mechanism of molecular vibration friction increases the contact between the alkali solution and chitin molecular chain, thus allowing the β‐1,4 glycosidic bonds to be more easily broken and reducing the molecular weight.

**Figure 8:**
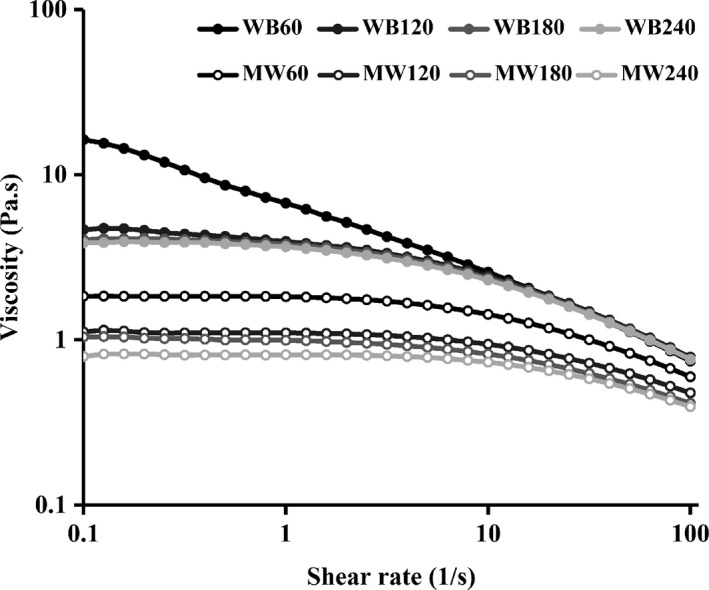
Effect of shear rates on the viscosity of chitosan produced by microwave heating and water bath heating

Viscosity is an important determinant for the applications of polymers, and the viscosity properties of chitosan produced by the two methods have also been studied. Since the deacetylation reaction required at least 60 min, the chitosan produced by the two methods could basically be dissolved in 1% acetic acid solution. Therefore, we investigated the rheological properties of chitosan that reacted for 60–240 min. All of the chitosan solutions present pseudoplastic fluid characteristics of shear thinning (Figure 8), which was due to the disassembly of macromolecular aggregation under external forces (Silva‐Weiss, Bifani, Ihl, Sobral, & Gómez‐Guillén, 2013). The configuration of the macromolecules then changed, and the molecules moved from disorder to order. The absence of the first Newtonian plateau in WB60 was due to the relatively higher M_W_ and the lower DD. MW240 and WB240 had a similar DD, the difference in their viscosity was mainly determined by the M_W_. As the chitosan with larger M_W_ had longer molecular chains, and the relative movement resistance between molecules was larger, the viscosity was generally higher (Wang, Guo, & Li, 2016), so the zero‐shear viscosity of MW240 was lower than that of WB240. In addition to the difference in M_W_ and DD, the electromagnetic field of MW may change the distribution of charge in chitosan molecules, making the rotation radius of chitosan smaller, resulting in a more oriented product with a lower viscosity.

### 3.5 Scanning electronic microscopy

**Figure 9:**
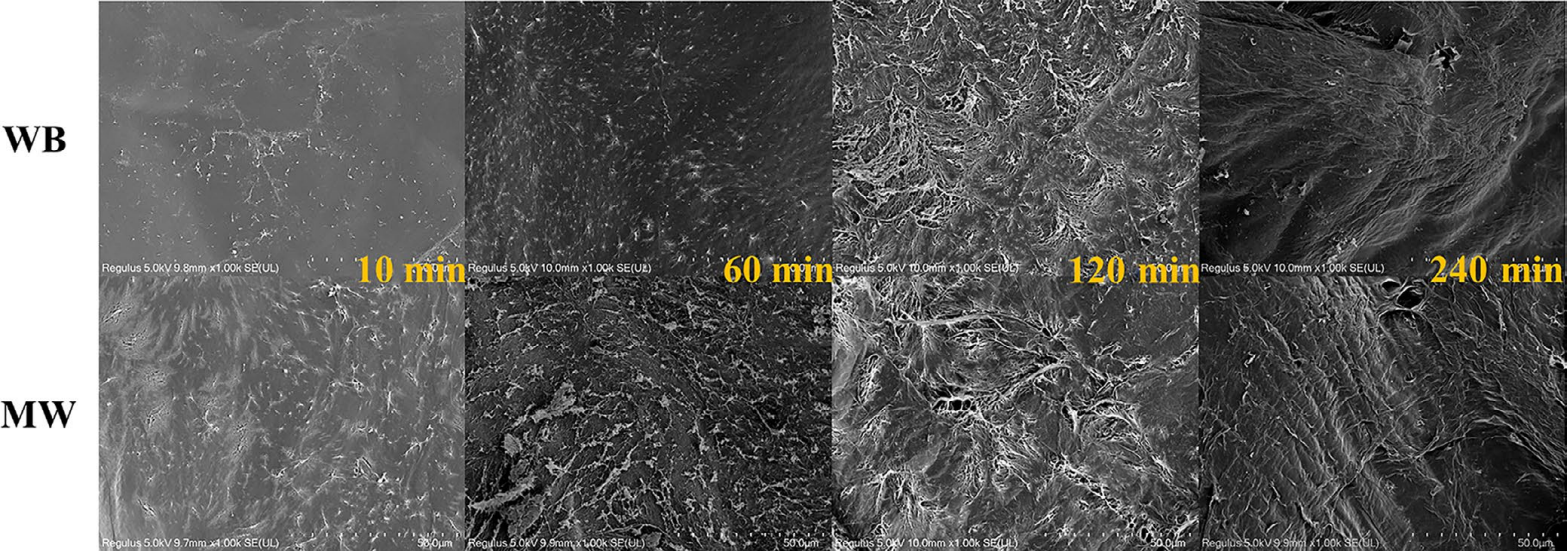
*SEM* photographs of chitosan produced by microwave heating and water bath heating at different reaction times

The morphology of chitosan produced by MW heating and WB heating was determined by *SEM*. The *SEM* photographs of the chitosan samples are shown in Figure 9. At the beginning of the reaction (10 min), deacetylation was carried out from the surface of the samples and then gradually extended into the interior. After a long time (240 min) of treatment with thermal aqueous alkali, the samples were basically deacetylated, the chitosan fibers were rearranged after swelling, the surface morphology showed a tight arrangement of fiber bundles, and the holes in the early stage disappeared as well. According to the *SEM* photographs after 10 min of reaction, MW significantly accelerated the rate of the deacetylation reaction based on the presence of more fiber breaks and holes, which was consistent with the trend of the DD value and verified the high efficiency of the special heating mechanism of MW for the deacetylation reaction.

## 4 CONCLUSION

Chitosan was successfully produced using the MW and WB heating methods at the same heating rate for the first time. Chitosan production with MW heating reduced the time of deacetylation from 180 min to 60 min to reach the same DD% as the traditional method with the same quantity of heat (DD% chitosan MW = 73.86% and DD% chitosan WB = 74.47%). While the structure and morphology of chitosan MW were similar to those of chitosan WB, chitosan MW also proved to have higher crystallinity and lower M_W_ and zero‐shear viscosity than chitosan WB. These results showed that the contact between the solid and liquid was enhanced by the molecular vibration in MW heating, which made the alkali solution more accessible to the chitin and promoted deacetylation. This study contributes to the better understanding of the differences in deacetylation process heated by MW and traditional heat source, as well as the selection of heat sources and heating procedures for the production of chitosan for different application requirements, which could improve the consistency of the product quality.

## CONFLICTS OF INTEREST

There are no conflicts of interest to declare.

## ETHICAL APPROVAL

This study does not involve any human or animal testing.
